# Type I Interferon Regulates the Expression of Long Non-Coding RNAs

**DOI:** 10.3389/fimmu.2014.00548

**Published:** 2014-11-06

**Authors:** Elena Carnero, Marina Barriocanal, Victor Segura, Elizabeth Guruceaga, Celia Prior, Kathleen Börner, Dirk Grimm, Puri Fortes

**Affiliations:** ^1^Department of Gene therapy and Hepatology, Center for Applied Medical Research (CIMA), University of Navarra, Pamplona, Spain; ^2^Bioinformatics Unit, Center for Applied Medical Research (CIMA), University of Navarra, Pamplona, Spain; ^3^Centre for Infectious Diseases/Virology, Heidelberg University Hospital, Cluster of Excellence CellNetworks, Heidelberg, Germany; ^4^German Center for Infection Research (DZIF), Heidelberg, Germany

**Keywords:** IFN, lncRNAs, HCV, HIV, viral infection, IRF1, GBP1

## Abstract

Interferons (IFNs) are key players in the antiviral response. IFN sensing by the cell activates transcription of IFN-stimulated genes (ISGs) able to induce an antiviral state by affecting viral replication and release. IFN also induces the expression of ISGs that function as negative regulators to limit the strength and duration of IFN response. The ISGs identified so far belong to coding genes. However, only a small proportion of the transcriptome corresponds to coding transcripts and it has been estimated that there could be as many coding as long non-coding RNAs (lncRNAs). To address whether IFN can also regulate the expression of lncRNAs, we analyzed the transcriptome of HuH7 cells treated or not with IFNα2 by expression arrays. Analysis of the arrays showed increased levels of several well-characterized coding genes that respond to IFN both at early or late times. Furthermore, we identified several IFN-stimulated or -downregulated lncRNAs (ISRs and IDRs). Further validation showed that ISR2, 8, and 12 expression mimics that of their neighboring genes GBP1, IRF1, and IL6, respectively, all related to the IFN response. These genes are induced in response to different doses of IFNα2 in different cell lines at early (ISR2 or 8) or later (ISR12) time points. IFNβ also induced the expression of these lncRNAs. ISR2 and 8 were also induced by an influenza virus unable to block the IFN response but not by other wild-type lytic viruses tested. Surprisingly, both ISR2 and 8 were significantly upregulated in cultured cells and livers from patients infected with HCV. Increased levels of ISR2 were also detected in patients chronically infected with HIV. This is relevant as genome-wide guilt-by-association studies predict that ISR2, 8, and 12 may function in viral processes, in the IFN pathway and the antiviral response. Therefore, we propose that these lncRNAs could be induced by IFN to function as positive or negative regulators of the antiviral response.

## Introduction

Transcriptome analysis by tiling arrays and RNA sequencing has led to the conclusion that while 70–90% of the genome is transcribed, only 2% is dedicated to the transcription of protein-coding sequences (1, 2). Among the non-coding transcriptome, there is a group of poorly studied transcripts longer than 200 nt and with low coding potential that have been collectively called long non-coding RNAs (lncRNAs) (1, 3). It has been estimated that there could be as many lncRNA genes as coding genes, but the number of lncRNAs is still growing and some authors consider that it could increase to up to ~200000 (4, 5). Therefore, there is a great need to identify novel lncRNAs and to understand their function and regulation.

Long non-coding RNAs genes are very similar to coding genes at the chromatin, DNA, and RNA level (6). Compared to mRNAs, most lncRNAs are more cell-type specific, less expressed, and less conserved at the nucleotide sequence level (7). Many lncRNAs have been shown to be functional. Some lncRNAs function to regulate the expression of neighboring or antisense genes by transcriptional interference, by recruitment of chromatin modifiers and remodelers, or by regulation of imprinting, editing, splicing or translation, and stability (8–12). Enhancer RNAs (eRNAs) and lncRNA-activating RNAs (lncRNA-a) are transcripts that control the expression of neighboring genes in “cis” (13–15). However, lncRNAs can also function in “trans,” away from their site of synthesis. For instance, some pseudogenes regulate the expression of their parental gene, located in a distant genomic location (16–19). LncRNAs have especially emerged as regulators of development, pluripotency, and proliferation as some function as oncogenes or tumor suppressors (6, 12, 20–23). Therefore, several lncRNAs have been implicated in cancer and in other human diseases (24–26).

Proliferation, differentiation, and pluripotency factors regulate the expression of some lncRNAs (27). Besides, several signaling molecules, including those involved in the immune response, have been shown to induce the expression of specific lncRNAs (28–31). Induction of TLR2, TLR3, or TLR4 leads to the activation of lncRNAs, including lncRNA-COX2, which regulates the expression of several immune genes or NEAT1, which functions to increase the expression of some antiviral genes such as IL8 (32–34). Downregulation of IL1β-eRNA and IL1β-RBT46 lncRNAs decreases IL1β and the accumulation of LPS-induced RNAs (34). Similarly, downregulation of lnc-IL7R decreases the LPS-induced inflammatory response (35). Treatment of THP1 macrophages with an innate immunity activator also induces the expression of several lncRNAs. One of them, linc1992 (or THRIL) activates the expression of TNFα and other genes involved in the immune response (36). In turn, TNFα also induces many lncRNAs in fibroblasts, including Lethe, a pseudogene that responds to NFκB and inhibits NFκB DNA-binding activity leading to reduced inflammation (37). Besides, dendritic cells (DCs), CD4+, and CD8+ T-cells express a specific set of lncRNAs that may regulate cell activation and differentiation (6, 38, 39). NEST lncRNA controls the IFNγ locus in CD8+ T-cells causing decreased *Salmonella enterica* pathogenesis (40, 41). Downregulation of lnc-DC, expressed in conventional DCs, impairs DC differentiation from monocytes, and reduces the capacity of DCs to activate T-cells (42). LncRNAs also respond to viral infections. Infection with enterovirus, influenza virus, HIV, hepatitis B, and C (HCV) viruses as well as the SARS coronavirus leads to altered levels of lncRNAs (33, 43–50) (Carnero et al., in prep). From the collection of infection-altered lncRNAs, it is difficult to distinguish those that respond to the virus from those that respond to the cellular antiviral pathways activated by the infection. Recently, some lncRNAs regulated by infection have also been found to be regulated by IFNα in mice (46).

Interferon is a key molecule in the cellular antiviral response (51). Detection of pathogens by the cell triggers transcription of the interferon (IFN) genes. When type I/III IFN is released, it is sensed by the IFN receptors, which induce the JAK/STAT pathway. STAT1 and 2 coupled to IRF9 form a complex that binds IFN-stimulated response elements (ISRE) in the promoters of IFN-stimulated genes (ISGs) and activates their transcription. ISGs induce an antiviral state by several means, including inhibition of viral replication, transcription, and translation. Well-characterized ISGs are Mx1, OAS, GBP1, but also STAT1 and IRF9, which amplify the IFN response. Further, STAT1 induces the expression of pro-inflammatory genes such as IRF1, a transcription factor that also activates ISGs, and whose induction is dependent on *de novo* protein synthesis (52, 53). Besides, IFN also induces the expression of negative regulators that limit the strength and duration of the IFN response (54–56). Finally, IFN activates expression of several miRNAs that contribute to the antiviral state or to the control of the IFN response (57).

Here, we have postulated that IFN could also regulate the expression of lncRNAs that may have key roles in the antiviral response. Therefore, we have performed a high-throughput analysis of lncRNAs whose expression is deregulated in response to IFNα. The conditions we used aimed to identify genes controlled by the IFN pathway directly or by other ISGs. The results show that several lncRNAs are controlled in response to type I IFN in several cell lines tested. The best candidates are lncRNA genes upregulated in response to IFN that are found in the genome adjacent to IFN-related coding genes. They have been called ISR2, 8, and 12. Interestingly, guilt-by-association genome-wide studies predict that the function of these lncRNAs is related to the cellular antiviral response and to viral infections. In fact, ISR2, 8, and their neighboring genes are also increased after infection of cultured cells with HCV. A similar increase is detected in the livers of patients infected with this virus or, in the case of ISR2, in blood cells of patients infected with HIV.

## Materials and Methods

### Cells and patient samples

HuH7 cells, derived from a human hepatocarcinoma, were provided by Dr. Chisari’s lab (Scripps Research Institute, La Jolla, CA, USA). A549 and THP1 cells were kindly provided by Estanislao Nistal (CIMA, University of Navarra, Spain), and HeLa and 293 cells were obtained from ATCC. Liver samples from patients with or without HCV infection were obtained from the Biobank of the University of Navarra under approval from the Ethics and Scientific Committees. Liver tissue sections were snap frozen and stored at −80°C. The clinical data from HCV and HIV-infected subjects are shown in Table S1 and S2 in Supplementary Material.

### Cell culture

Cells were grown in Dulbecco’s Modified Eagle Medium (DMEM) enriched with 10% fetal bovine serum (FBS) and 1% penicillin–streptavidin in a 5% CO_2_ atmosphere. Twenty-four hours before treatment with IFN, HuH7, A549, THP1, 293, or HeLa cells were seeded in six-well plates. Then, 0, 5, 50, 250, 1000, or 10000 u/ml of IFNα2 (Sicor Biotech) or IFNβ (PBL Pestka Biomedical Laboratories) were used in a final volume of 2 ml. HuH7 cells were also treated with 250 ng/ml IL28B/IFN-λ3 (R&D Systems) in a final volume of 2 ml. Cells were harvested for RNA extraction 6, 12, 24, 48, and/or 72 h after treatment.

### Viral infections

HCV JFH-1 was obtained from an initial viral stock from the genotype 2a JFH-1 plasmid (pJFH-1) previously described by Wakita et al. (58). To amplify the virus, HuH7 cells were infected at low multiplicity of infection (moi) with the initial viral stock and supernatants from the cells were harvested at different days post-infection. The presence of virus was evaluated by infecting fresh cells with the supernatant and checking infected cells by immunofluorescence against the HCV core protein. The supernatants with higher titers were selected to perform the experiments. Influenza virus strain A/PR8/34 WT (PR8) and the mutant lacking NS1 (ΔNS1) were kindly provided by Estanislao Nistal (CIMA, University of Navarra, Spain) (59), Semliki Forest Virus (SFV) was a gift from Cristian Smerdou (CIMA, University of Navarra, Spain), and Adenovirus serotype 5 (Ad5) was amplified as described (60). Twenty-four hours before infection, cells were seeded in six-well plates in a final volume of 2 ml. Cells were infected with HCV at a moi of 0.3, and with a moi of 10 of Influenza A, ΔNS1, Ad5, and SFV. In the case of the lytic viruses, we used a moi of 10 as this led to cytopathic effects at 24 h (for Influenza and SFV) or 48 h (for Ad5) in HuH7 cells. Infection with HCV was performed for 4 h, versus 2 h in the case of Ad5 and 1 h in the case of the other viruses. A final volume of 1 ml was used for infection. After infection, the virus was removed and fresh medium was added to the cells. Cells were harvested for RNA extraction at the indicated times post-infection.

### Cellular fractionation

Two million HuH7 cells were incubated in 100 μl of cytoplasmic buffer (50 mM Tris HCl pH7.4, 1 mM EDTA, and 1% NP40) for 5 min at 4°C. Then, cells were centrifuged for 5 min at 3000 *g* and the supernatant was used to isolate cytoplasmic RNA. The pellet was washed with cytoplasmic buffer and centrifuged as before. The supernatant was discarded and the pellet was used to isolate the nuclear RNA. RNA from nuclear and cytoplasmic fractions was isolated with MaxWell 16 research system (Promega).

### RNA extraction and microarray hybridization

Total RNA from tissue samples was extracted in 1 ml TRIZOL (Sigma-Aldrich) using the ULTRA-TURRAX homogenizer (t25 basic IKA-WERKE) (61). Then, 200 μl chloroform was added and the samples were mixed vigorously and then centrifuged at 12000 *g* for 15 min at 4°C. The aqueous phase was mixed with 800 μl isopropanol and centrifuged at 12000 *g* for 10 min at 4°C. The pellet of total RNA obtained from the centrifugation was washed with 70% ethanol. Finally, the pellet was resuspended in 30 μl DNase/RNase-free PCR water (Bioline). DNase I (Fermentas) treatment was performed to eliminate DNA from the samples before the reverse transcription (RT)–PCR reactions.

To isolate RNAs from total blood, 2.5 ml blood were collected into PAXgene Blood RNA tubes with RNA stabilization solution. Total RNA was extracted by using the PAXgene Blood RNA kit (Qiagen GmbH) according to the manufacturer’s instructions. Briefly, prior to the actual RNA isolation, the frozen samples were first incubated at RT for at least 2 h to achieve complete lysis of blood cells. Then, the PAXgene Blood RNA tubes were centrifuged for 10 min at 3000 *g*, the supernatant was removed and the pellet was washed with 4 ml RNase-free water. The pellet was then dissolved in 350 μl of the provided lysis buffer BM1 and transferred into a 1.5 ml microcentrifuge tube. To this mixture, 300 μl buffer BM2 and 40 μl proteinase K were added and incubated for 10 min at 55°C in a shaking incubator at 1000 rpm. The samples were next transferred to a PAXgene Shredder spin column and centrifuged for 3 min at full speed. The flow-through was transferred into a new tube without disturbing the pellet and mixed with 700 μl isopropanol (100%, purity grade p.a.). This mixture was then passed through a PAXgene RNA spin column by centrifugation for 1 min at 8000 *g*. Following a wash step (350 μl BM3), an on-column DNase digest (RNase-Free DNase Set, Qiagen) was performed by addition of DNase I and incubation on the benchtop (20–30°C) for 15 min. The column was washed three times, before RNA was eluted with 80 μl buffer BR5. The final eluate was incubated for 5 min at 65°C, and several aliquots of the RNA were stored at −80°C.

RNA extraction from cells or cellular fractions was performed using the MaxWell 16 research system from Promega according to the manufacturer’s recommendations. For each condition, a minimum of a confluent well of a M6-plate was used in order to obtain enough RNA. In all cases, the RNA concentration was measured using a NanoDrop 1000 Spectrophotometer. The quality of the RNA was determined in a Bioanalyzer (Agilent technologies). For microarray hybridization, the samples were processed using manufacturer protocols and hybridized to the Agilent SurePrint G3 Human Gene Expression 8 × 60 K microarray. Transcriptome data are available at the NCBI Gene Expression Omnibus (GEO) data repository^1^.

### Quantitative polymerase chain reaction

Reverse transcription was performed using 1.2 μl M-MLV-RT and 8 μl M-MLV-RT 5× buffer (Promega), 4 μl 5 mM dNTPs, 4 μl Random Primers at 100 ng/μl, 2 μl DTT 0.1 M, and 1 μg RNA in a final volume of 40 μl. The reaction was run in the C1000 Touch Thermal Cycler from Bio-Rad. The samples were incubated at 37°C for 60 min, then at 95°C for 60 s, and next immediately placed at 4°C.

Quantitative polymerase chain reaction was performed in the CFX96 Real-Time system from Bio-Rad. For the reaction, 10 μl IQ Syber Green mix from Bio-Rad, 0.4 μl of each primer at 15 μM, and 2 μl of the DNA sample at 0.01 μg/μl were mixed in a final volume of 20 μl. The mixture was first incubated at 95°C for 3 min, and then at 95°C for 15 s, 60°C for 15 s, and 72°C for 25 s for 34 cycles. The PCR ended after 1 min at 95°C and 1 min at 65°C. The results were analyzed with Bio-Rad CFX-manager software. GAPDH levels were evaluated in all cases as a reference. Only the samples with similar GAPDH amplification were analyzed further. The primers used are listed in Table S3 in Supplementary Material and were designed using the Primer3 program^2^. Initial setups included GC percentage between 30 and 70%, product size from 150 to 300 bp and primer length between 18 and 27 nt.

### Bioinformatic and statistical analysis

Microarray data normalization was performed using the quantile algorithm. After quality assessment, a filtering process was carried out to eliminate low expression probe sets. Applying the criterion of an expression value >64 in the three samples of at least one of the experimental conditions, 45322 probe sets were selected for statistical analysis. LIMMA (Linear Models for Microarray Data) (62) was used to identify the probe sets with significant differential expression between experimental conditions. Genes were selected as significant using a B statistic cut-off of *B* > 1.5. Data processing and statistical analyses were performed with R and Bioconductor (63).

Functional enrichment analysis of Gene Ontology (GO) categories was carried out using standard hypergeometric test (64). The biological knowledge extraction was complemented through the use of Ingenuity Pathway Analysis (Ingenuity Systems)^3^, whose database includes manually curated and fully traceable data derived from literature sources. All the differentially expressed sequences obtained by the analysis were compared to the ENSEMBL and ENCODE databases and searched for in the Genome Browser from UCSC^4^ for more information (65, 66). ORF Finder (NCBI) was used to evaluate the length of all probable ORFs in ISR2, 8, and 12. Coding potential was assayed with the coding potential assessment tool (CPAT) (67, 68) and by searching the LNCipedia database (69) for the presence of our candidates in the Pride archive (70) or in lists of transcripts associated with ribosomes (71, 72). Phylogenetic Codon Substitution Frequencies (PhyloCSF) was also used to predict the coding potential of ISR2, 8, and 12 (73).

A guilt-by-association approach was used to predict the GO categories (74) in which the differentially expressed lncRNAs could be implicated. First, we collected data from 120 samples hybridized to SurePrint G3 microarrays. These samples include the 6 RNAs isolated from HuH7 cells treated or not with IFN, and 114 RNAs obtained from human samples of different origin, including healthy tissues and several leukemias and other tumors. Then, a Pearson correlation analysis was performed between ISR2, 8, and 12 and all the genes represented in the SurePrint G3 Human microarray. Coding genes related to cellular antiviral pathways were randomly selected and included in the analysis as positive controls. The obtained correlation matrix was used as input for giTools (75) where an enrichment analysis of GO categories was performed using *Z*-score (76) and FDR (77).

Statistical analysis of the expression levels obtained by quantitative RT-PCR (qRT-PCR) was performed using graph-path. Statistical significance of treated or infected versus non-treated or non-infected samples was calculated using a two-tailed non-parametric Mann–Whitney *t*-test. In correlation studies, a two-tailed non-parametric Spearman analysis was used. Similar results were obtained by Pearson correlation. *P* values lower than 0.05 were deemed as significant.

## Results

### High doses of IFNα induce the expression of several genes involved in the IFN response

**Figure 1:**
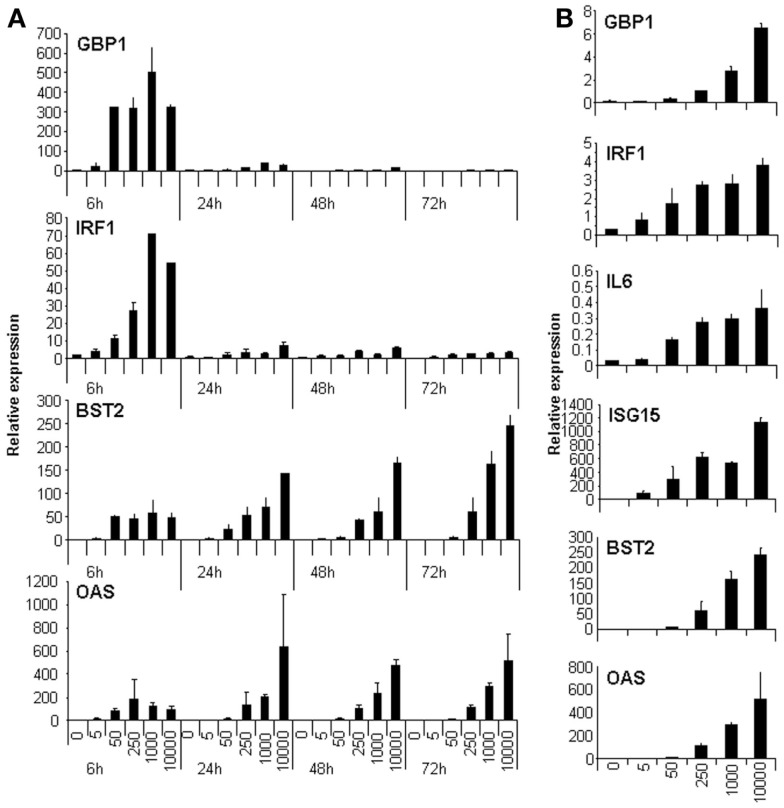
**High doses of IFN induce the expression of genes involved in the IFN response at late times post-treatment**. HuH7 cells were treated for 6, 24, 48, or 72 h **(A)** or for 72 h **(B)** with 0, 5, 50, 250, 1000, or 10,000 units/ml of IFNα2 and the expression levels of GAPDH, GBP1, IRF1, BST2, OAS (**A** and **B**), or of IL6 and ISG15 **(B)** were evaluated by qRT-PCR. The relative expression was calculated using GAPDH as a reference. The experiment was performed three times and each value shows the average of three replicas from a representative experiment. Error bars indicate standard deviations.

We wanted to identify lncRNAs that respond to IFN. IFN induces the expression of ISGs very fast. Some ISGs are transcription factors able to regulate the expression of genes with antiviral potential in a secondary wave of IFN response. Other ISGs are inhibitory factors that function to decrease the response. Therefore, to identify lncRNAs regulated by IFN or by ISGs, one should analyze the transcriptome of cells treated by IFN at different time points. However, to simplify the analysis, we decided to check first whether we could find conditions showing a wide IFN response at a single time point. To this aim, we tested whether high doses of IFN could lead to increased expression of well-known ISGs even at late times post-IFN treatment. HuH7 cells were treated for 6, 24, 48, or 72 h with increasing doses of IFNα2 up to 10,000 units/ml, and the expression levels of GBP1, IRF1, BST2, OAS, IL6, and ISG15 were evaluated by qRT-PCR (Figure 1). The results show that GBP1 and IRF1 are induced to highest levels at 6 h post-treatment, while BST2 and OAS are induced to similar levels at all times tested. In contrast, IL6 is only significantly induced at 3 days post-treatment (Figure 1 and data not shown). However, compared to untreated cells, a significant upregulation of all the genes, including GBP1 and IRF1, can be detected at 3 days post-treatment with 10,000 units/ml of IFNα2 (Figure 1B). In fact, this dose induced the highest levels of these transcripts at most of the time points. Before analyzing the transcriptome of cells treated with 10,000 units/ml of IFNα2 for 3 days, we confirmed that these conditions induced antiviral effects. When HuH7 cells infected with HCV were treated with these conditions, we indeed detected a drastic decrease in viral protein expression by immunofluorescence and a decrease in the levels of HCV viral genomes by qRT-PCR (data not shown).

### Identification of LncRNAs regulated by IFNα

**Figure 2:**
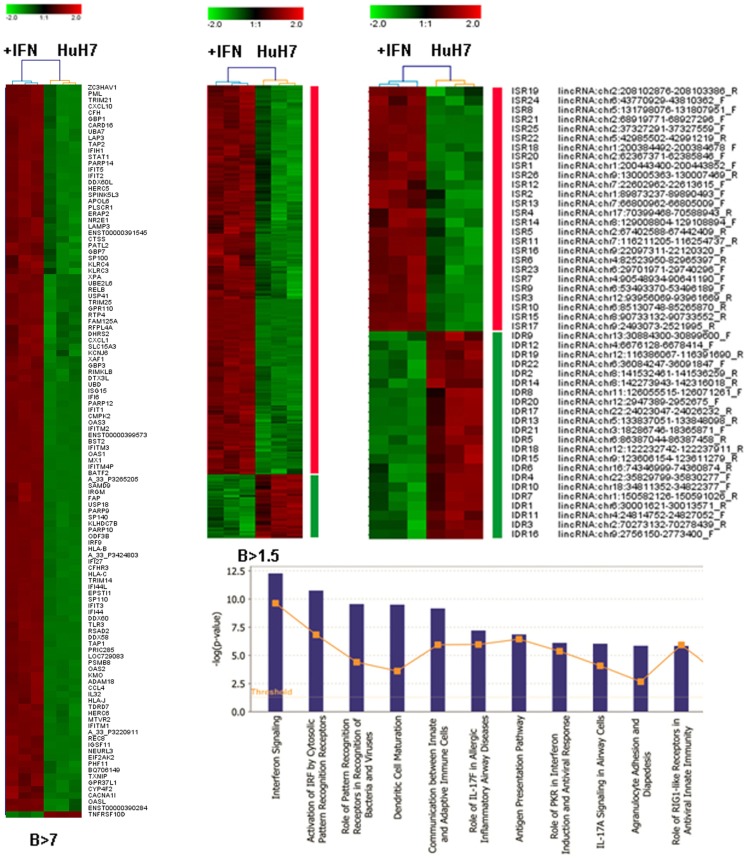
**Microarray analysis of samples treated with IFN**. HuH7 cells were treated for 72 h with 0 or 10,000 units/ml of IFNα2 in three independent experiments. RNA isolated from these cells was hybridized to an Agilent array that interrogates the expression of 27958 Entrez genes and 7419 lncRNAs. Heat map clustering of all genes with *B* > 7 **(A)**, *B* > 1.5 **(B)**, or the curated probes described as lincRNAs with *B* > 1.5 **(D)** is shown. Ingenuity analysis of the set described in B is also shown **(C)**. The color scale is indicated for each heat map and uses log 2 units. Upregulated probes are shown in red. Downregulated probes are shown in green.

An Agilent array that evaluates expression of 27958 Entrez genes and 7419 lncRNAs was used to hybridize RNA isolated in three independent experiments from control cells or HuH7 cultures treated with 10,000 units/ml of IFNα2 for 3 days. Analysis of the array showed that genes upregulated with a high statistical significance (*B* > 7) includes well-known IFN-related genes from the GBP, IFI, OAS, ISG, MX, or IRF families (Figure 2A). Analysis using less stringent criteria (*B* > 1.5) showed that 90% of the genes were upregulated in response to IFN treatment (Figure 2B). Ingenuity analysis of this set indicated that IFN signaling is the pathway with the highest enrichment followed by other antiviral responses (Figure 2C). Similarly, the IFN-induced STAT pathway and the TLR/IRF network are well represented in the set of upregulated genes (Figure S1 in Supplementary Material).

We selected the probes described as long intergenic non-coding RNAs (lincRNAs) that showed a significantly altered expression by the IFN treatment (*B* > 1.5). First, we determined the position in the genome of the sequences from these probes using the BLAT searches available at UCSC (78, 79). Many sequences corresponded to coding genes or seemed to be 3′UTR extensions of coding genes and were discarded for further analysis. The remaining sequences corresponded to 48 genes, including 29 genes annotated as lncRNAs and 19 located in non-annotated areas of the genome (Figure 2D, Table S4 in Supplementary Material). Surprisingly, while 90% of coding genes were upregulated by IFN, only 54% of the lncRNAs were upregulated. Most of the downregulated lncRNAs corresponded to the non-annotated category. This suggests that there could be a relevant IFN-mediated repression of genes that more strongly affects lncRNAs. We named these genes IDRs, for IFN-downregulated RNAs, and accordingly named the IFN-stimulated RNAs ISRs. The fact that almost 40% of the ISRs and IDRs correspond to non-annotated areas of the genome suggests that the percentage of the genome able to react to different stimuli could be even larger than expected.

As many lncRNAs have been described to regulate the expression of neighboring genes, we looked for the closest coding gene for each ISR or IDR (Table S4 in Supplementary Material). We considered candidates to have no neighbor when the closest coding gene was not within a distance of 100 kb from the start or the end of the candidate or when the closest gene was non-coding. Forty candidates had neighboring coding genes according to these criteria. Half of the coding-non-coding pairs were in tandem, convergent, or divergent, 12 were antisense to each other, 4 were overlapping, and 5 pairs seem to share the same promoter according to the DNase I hypersensitivity and the histone marks described by ENCODE for the respective area. Therefore, these couples of coding-non-coding genes could be co-regulated. Three upregulated candidates, ISR2, ISR8, and ISR12 were neighbors of the IFN-related genes GBP6, IRF1, and IL6, respectively.

### IFNα alters the expression of LncRNAs at different time points and in different cell lines

**Figure 3:**
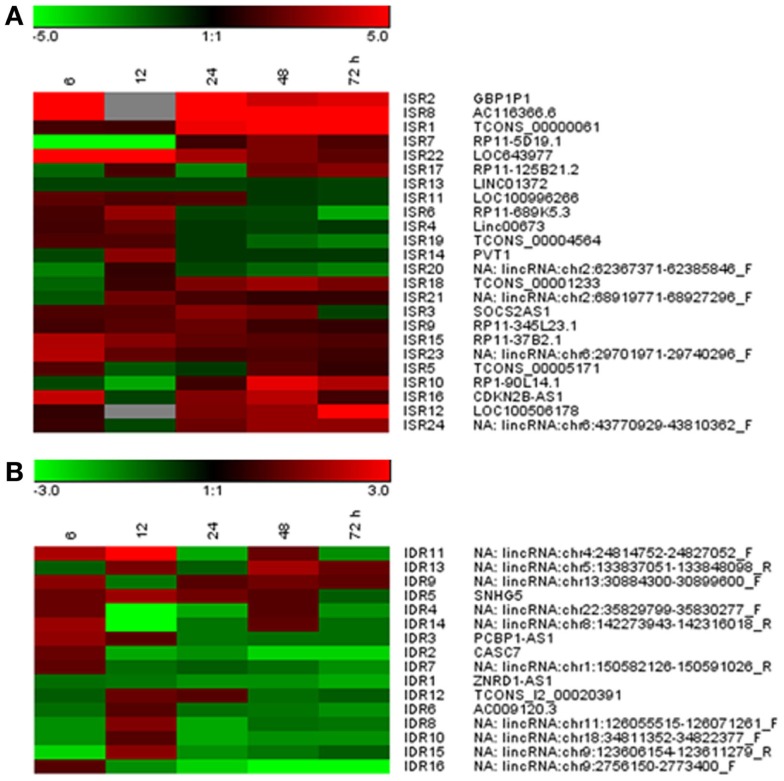
**Validation of IDRs and ISRs at different times after IFN treatment**. Expression levels of 24 ISRs **(A)** and 16 IDRs **(B)** were evaluated by qRT-PCR in HuH7 cells treated with 0 or 10,000 units/ml of IFNα for 6, 12, 24, 48, or 72 h. GAPDH was also evaluated by qRT-PCR and used as a reference to calculate the relative levels of each transcript. The ratio of levels with IFN versus no IFN is shown for each candidate at each time point. The results have been clustered and are shown in the form of a heat map. The color scale is shown at the top. Red denotes upregulation and green downregulation. A maximum of linear units from −5 to +5 **(A)** or −3 to +3 **(B)** has been set as indicated in the color scale.

We next wanted to validate the IFN effect on these candidates in independent samples using a different technique. Furthermore, we wanted to determine whether the levels of ISRs and IDRs were altered early after IFN treatment. Therefore, the expression levels of 24 ISRs and 16 IDRs were evaluated by qRT-PCR in HuH7 cells treated with 0 or 10,000 units/ml of IFNα for 6, 12, 24, 48, or 72 h. The fold-change observed for each candidate at each time point is shown in Figure 3 and Table S5 in Supplementary Material. At 72 h post-treatment, 11 ISRs and 8 IDRs showed a fold-change higher or lower, respectively, than 1.5. Only ISR13 and 20 were not significantly upregulated, or IDR3, 5, 9, 12, and 13 were not significantly downregulated, at any time tested. However, the fold-change was relatively low in most of the cases, indicating a weak response to IFN. Moreover, 40% of the candidates showed low overall expression levels (Table S4 in Supplementary Material and data not shown). Interestingly, ISR2 and ISR8 were induced more than 50-fold at 6 h post-IFN treatment, and ISR1 was induced more than 20-fold at later times. Therefore, we decided to study these candidates further. We also opted to focus on ISR10 and 12, as they were upregulated at later time points. IDR1 and IDR2, which were downregulated at most times studied, were also analyzed further.

**Figure 4:**
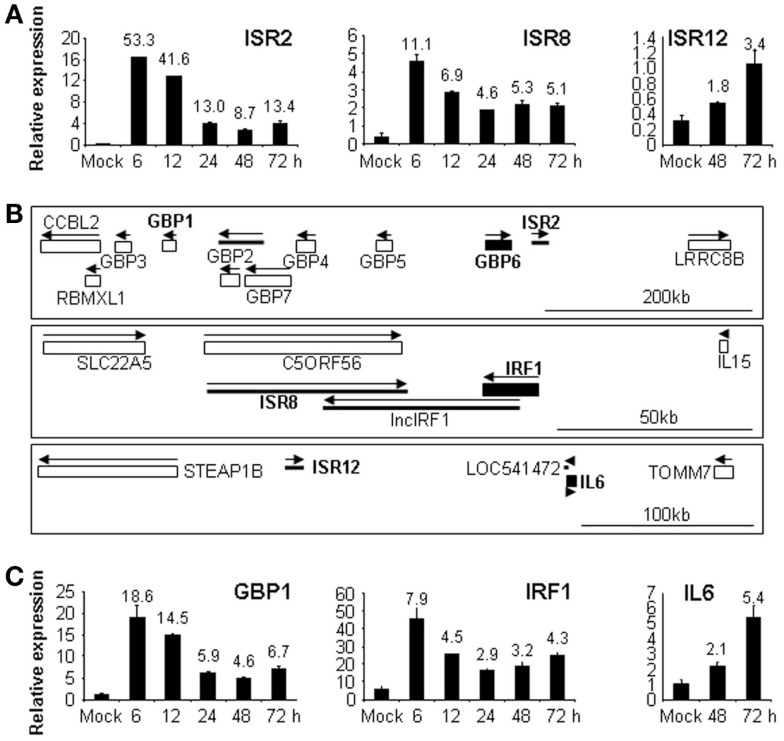
**ISR2, 8, and 12 and their neighboring coding genes respond to IFN in different cell lines**. A549 or HeLa cells were treated with 0 or 10,000 units/ml of IFNα for the indicated times and RNA was isolated and used to evaluate the expression of ISR2, 8 [**(A)**, A549], ISR12 [**(A)**, HeLa] or their neighboring genes GBP1, IRF1 [**(C)**, A549], and IL6 [**(C)**, HeLa]. GAPDH was also evaluated by qRT-PCR and used as a reference to calculate the relative levels of each transcript. The experiment was performed three times and each value shows the average of three replicas from a representative experiment. Error bars indicate standard deviations. The fold-change of IFN-treated versus non-treated samples is indicated at the top of each bar. **(B)** Schematic representation of the genomic location of ISR2, 8, and 12 and their neighboring genes taken from UCSC database. Coding genes are shown with rectangles and non-coding genes with thick lines. GBP6, IRF1, and IL6 are shown with filled rectangles and in bold. ISR2, 8, and 12 are also in bold. Arrows indicate sense and antisense orientation. The scale bar is shown at the bottom to the right.

As many lncRNAs are cell-specific, we decided to study the response to IFN of these selected ISRs/IDRs in different cell lines. HeLa, 293, A549, or THP1 cells were treated with 0 or 10,000 units/ml of IFNα for 6, 12, 24, 48, or 72 h and RNA was isolated and used to evaluate the expression of ISR1, 2, 8, 10, and 12 as well as IDR1 and 2. The results showed that in the new cell lines tested, expression of ISR1 and 10 was not detected and IDR1 and 2 showed only a mild downregulation in response to IFN (data not shown). Importantly, ISR2, 8, and 12 were well expressed in all cell lines tested and their expression was strongly induced by IFN at early (ISR2 and 8) or late times (ISR12) (Figure 4A and data not shown).

Interestingly, ISR2, 8, and 12 have neighboring genes related to IFN response (Figure 4B). ISR2 is located in tandem with GBP6, at the end of the cluster of GBP genes formed by GBP3, 1, 2, 7, 4, 5, and 6. In fact, ISR2 is GBP1P1, a pseudogene of GBP1. This may be interesting as some pseudogenes have been described to regulate the expression of their parental genes (16–19). ISR12 is located in tandem with IL6 and ISR8 is convergent with IRF1. Therefore, we decided to evaluate the expression of GBP1, IRF1, and IL6 in response to IFN in the same cell lines. The results showed a similar induction pattern of each ISR and of the corresponding neighboring coding gene in response to IFN (Figures 4A,C). Note that in the case of ISR2, we evaluated the expression of its parental neighboring gene GBP1 instead of the closest neighbor GBP6, as we speculated that there could be a co-regulation of the parental gene and the pseudogene. Furthermore, we could not detect expression of GBP6 in control or IFNα-treated HuH7 cells (data not shown).

**Figure 5:**
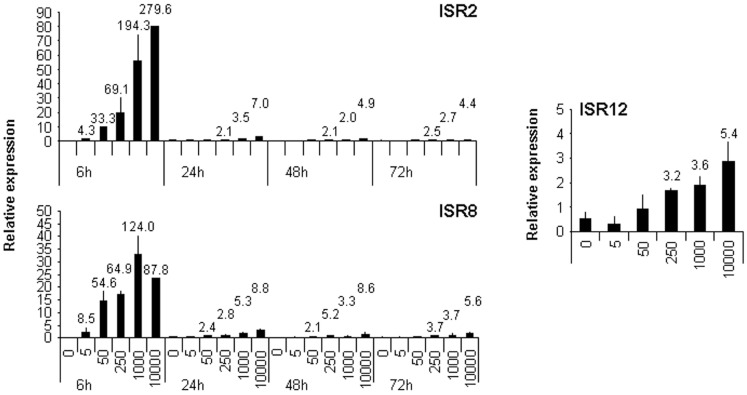
**ISR2, 8, and 12 respond to low doses of IFN**. HuH7 cells were treated for 6, 24, 48, or 72 h with 0, 5, 50, 250, 1000, or 10,000 units/ml of IFNα2 and the expression levels of ISR2 and 8 and ISR12 were evaluated by qRT-PCR. GAPDH expression was also evaluated and used as a reference to calculate the relative levels of each transcript. The experiment was performed three times and each value shows the average of three replicas from a representative experiment. Error bars indicate standard deviations. The fold-change of treated versus non-treated cells is indicated at the top of each bar when higher than two.

All the experiments performed so far with these lncRNAs have studied the response to high doses of IFN. To determine whether ISR2, 8, and 12 also respond to lower doses, their expression level was evaluated in HuH7 cells treated for 6, 24, 48, or 72 h with 5, 50, 250, 1000, or 10,000 units/ml of IFNα2 (Figure 5). The results show that induction of these lncRNAs is similar to that observed for their corresponding neighboring coding genes (compare Figure 5 with Figure 1). Similar results were observed when IFNβ was used instead of IFNα2 (data not shown). Further, we evaluated whether expression of these transcripts was induced after treatment with TNFα. TNFα, similarly to some pathogen-associated molecular patterns, induces NFκβ signaling and expression of pro-inflammatory genes. However, the NFκβ pathway is a poor inducer of ISGs. A treatment of HuH7 cells with 20 ng/ml of TNFα for 6 h, induced the expression of CXCL10, used as a positive control, more than 100-fold. A significant increase in expression of only 2–3-fold was observed after TNFα treatment for GBP1, IRF1, IL6, and ISR12, but not for ISR2. Similar results were obtained in HuH7 cells treated with LPS or polyI:C (data not shown).

To obtain more information about these ISRs, we looked in detail at the data from ENCODE. Even though we did not observe a strong activation after TNFα treatment, transcription factor chIP Seq from ENCODE showed that ISR8 and ISR12 promoters have NFκB binding sites. Interestingly, the ISR8 promoter has sites for STAT1 and 2 as well as IRF1 and 2, suggesting that ISR8 could be a *bona fide* ISG. Besides the non-coding transcript that we name ISR8, six other transcripts could be expressed from the ISR8 area according to UCSC and Ensembl databases (Figure S2A in Supplementary Material). These transcripts have certain coding capacities and could be translated to proteins of up to 126 amino acids named C5ORF56. The syntenic region in the mouse also comprises non-coding transcripts together with transcripts with certain coding potential that lead to peptides no longer than 32 amino acids (data not shown). Only five amino acids in the N-terminal part of the putative protein predicted in mouse are conserved in human. Given the poor conservation and the short size of the predicted peptides all the transcripts from this mouse region could be classified as non-coding (80, 81). To analyze the expression of ISR8 and all the putative coding transcripts annotated in the human ISR8 area, we used qRT-PCR. The results show that most of the transcripts are poorly detected in HuH7 or HeLa cells treated or not with IFN. The highest expression is observed for the ISR8 lncRNA (Figure S2B in Supplementary Material).

Finally, UCSC database also shows that ISR8 is convergent to IRF1 and antisense to a longer IRF1 transcript with poor coding capacity that we named lncIRF1 (Figure 4B, Figure S2A in Supplementary Material). If expressed, lncIRF1 could regulate the level of ISR8 via antisense mechanisms. Therefore, we have evaluated the expression of lncIRF1 in response to IFN. The results show that lncIRF1 is expressed and its levels are induced at short times after IFN treatment (Figure S2C in Supplementary Material). Unlike ISR8, ISR2, or ISR12 transcripts did not overlap with annotated transcripts from GBP6 or IL6 (Figure S3 in Supplementary Material).

### Analysis of the coding potential of ISR2, 8, and 12

We evaluated the coding capacity of ISR2, 8, and 12 bioinformatically. ORF Finder (NCBI) was used to determine all possible open reading frames in these ISRs (Figure S4A in Supplementary Material). The analysis shows that all putative ORFs are shorter than 100 aa. Then, we evaluated their coding potential with the CPAT (67, 68) (Figure S4B in Supplementary Material). CPAT uses a model built with open reading frame size and coverage together with codon (Ficket score) and hexamer (hexamer score) usage bias. According to this program, ISR2, 8, and 12 are non-coding as they have a coding probability much lower than 0.364, used as a threshold with the highest sensitivity and specificity to differentiate between coding and non-coding transcripts in humans (68). ISR2, 8, and 12 were also described as non-coding in LNCipedia (69). This lncRNA database shows that ISR2, 8, or 12 are not found in the Pride archive, a database for proteomic data, or in lists of transcripts associated to ribosomes in ribosome profiling experiments (70–72). ISR8 and ISR12 were also described as non-coding by the analysis of PhyloCSF, which uses multiple alignments to calculate the phylogenetic conservation score and determines whether a multi-species nucleotide sequence alignment is likely to represent a protein-coding region (73).

**Figure 6:**
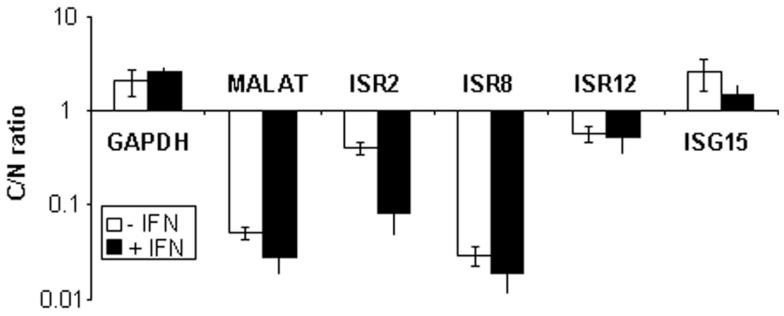
**ISR2, 8, and 12 accumulate preferentially in the nucleus**. HuH7 cells were mock-treated or treated with 10,000 units/ml of IFNα2 and divided into nuclear and cytoplasmic fractions. RNA was isolated from each fraction and used to evaluate the expression levels of ISR2, 8, and 12 by qRT-PCR. MALAT1, GAPDH, and ISG15 mRNA was also quantified and used as a reference to calculate the relative levels of each transcript and as a control to evaluate the subcellular fractionation. The ratio of cytoplasmic to nuclear levels is shown. The experiment was performed three times and each value shows the average of three replicas from a representative experiment. Error bars indicate standard deviations.

Finally, we evaluated the subcellular localization of ISR2, 8, and 12 in HuH7 cells mock-treated or treated with 10,000 units/ml of IFNα. RNA was isolated from nuclear or cytoplasmic fractions and quantified by qRT-PCR. The results show that the coding GAPDH or ISG15 mRNAs accumulate preferentially in the cytoplasm while the nuclear lncRNA MALAT1 is preferentially nuclear (Figure 6). Similarly, ISR2, 8, and 12 accumulate preferentially in the nucleus. This result, together with the bioinformatic analyses, strongly suggests that ISR2, 8, and 12 are non-coding RNAs.

### Prediction of ISR2, 8, and 12 function

**Figure 7:**
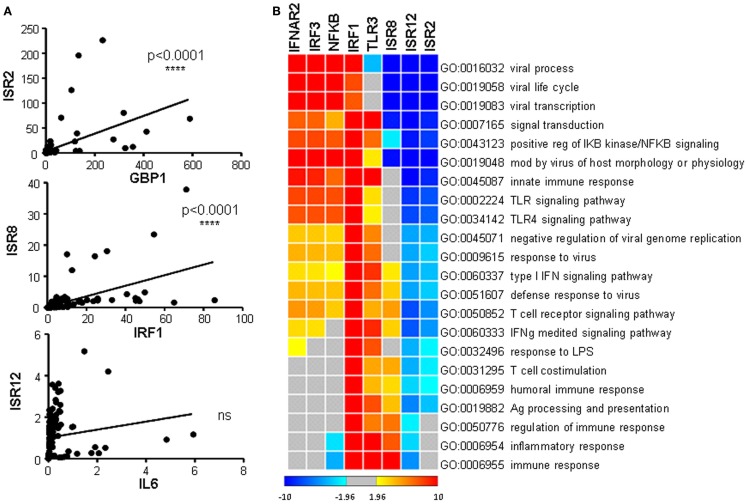
**Correlation of expression between ISR2, 8, and 12 and their coding partners, and guilt-by-association analysis**. **(A)** Expression levels observed for ISR2, 8, and 12 in Figures 4A and 5 were compared to the expression levels of their coding neighbors GBP1, IRF1, and IL6, respectively, in Figures 1 and 4C. A correlation analysis was performed and statistical significance was calculated using a two-tailed non-parametric Spearman analysis. **(B)** Clustering of the guilt-by-association results showing significant GO terms. The *z*-score color scale is shown at the bottom of the image.

As indicated above, each ISR and its neighboring coding gene have similar induction patterns in response to IFN (compare Figure 1 and Figure 5 or Figures 4A,C). This suggests that they could be co-regulated and therefore, that they could share similar functions. To analyze in more detail whether the expression level of each ISR correlates significantly with the expression level of its neighboring coding gene, we performed correlation studies. We compared the levels of each coding/non-coding pair in all the samples evaluated in Figures 1, 4, and 5. The results show a highly significant positive correlation between ISR2 and GBP1 or ISR8 and IRF1. In contrast, ISR12 had a non-significant correlation with IL6 (Figure 7A). Expression of neither ISR2 nor ISR8 significantly correlated with the expression of other ISGs such as OAS or BST2 (data not shown).

Arguably, this correlation analysis has been done with few ISGs and using homogeneous samples. Therefore, we decided to perform a more stringent high-throughput analysis of correlation. Accordingly, we carried out a guilt-by-association genome-wide analysis (74), which also predicts the function of unknown genes with high statistical confidence (Figure 7B). We compared the expression levels of ISR2, 8, and 12 and coding genes related to cellular antiviral pathways, used as positive controls, with the expression levels of all the genes represented in a SurePrint G3 microarray. We used data obtained from microarray experiments performed with 120 human samples of different origin. The results show a significant positive correlation between ISR8 and IRF1 (corr = 0.41 and *p* < 10e−0.5), indicating that these genes are co-regulated. Significant correlations were not observed for GBP1 and ISR2 or IL6 and ISR12. Furthermore, the correlation analysis of each candidate organized all the microarray genes from the ones with the highest positive correlation to the ones with the highest negative correlation. This matrix was used to search for GO categories with highly significant enrichment in genes that correlate positively (positive *z*-score) o negatively (negative *z*-score) with ISR2, 8, or 12. This analysis revealed that ISR2, 8, and 12 clustered very closely but away from other genes related with the IFN pathway and the antiviral response. ISR2, 8, and 12 showed a negative correlation with genes that significantly enriched GO categories related to viral processes including viral life cycle and viral transcription. ISR2 and 12 also shared a negative correlation with response to viral infection and IFN pathway genes. However, ISR8, similar to IRF1 and TLR3, showed a positive correlation with IFN signaling and immune response genes.

### ISR2 and ISR8 respond to viral infections

**Figure 8:**
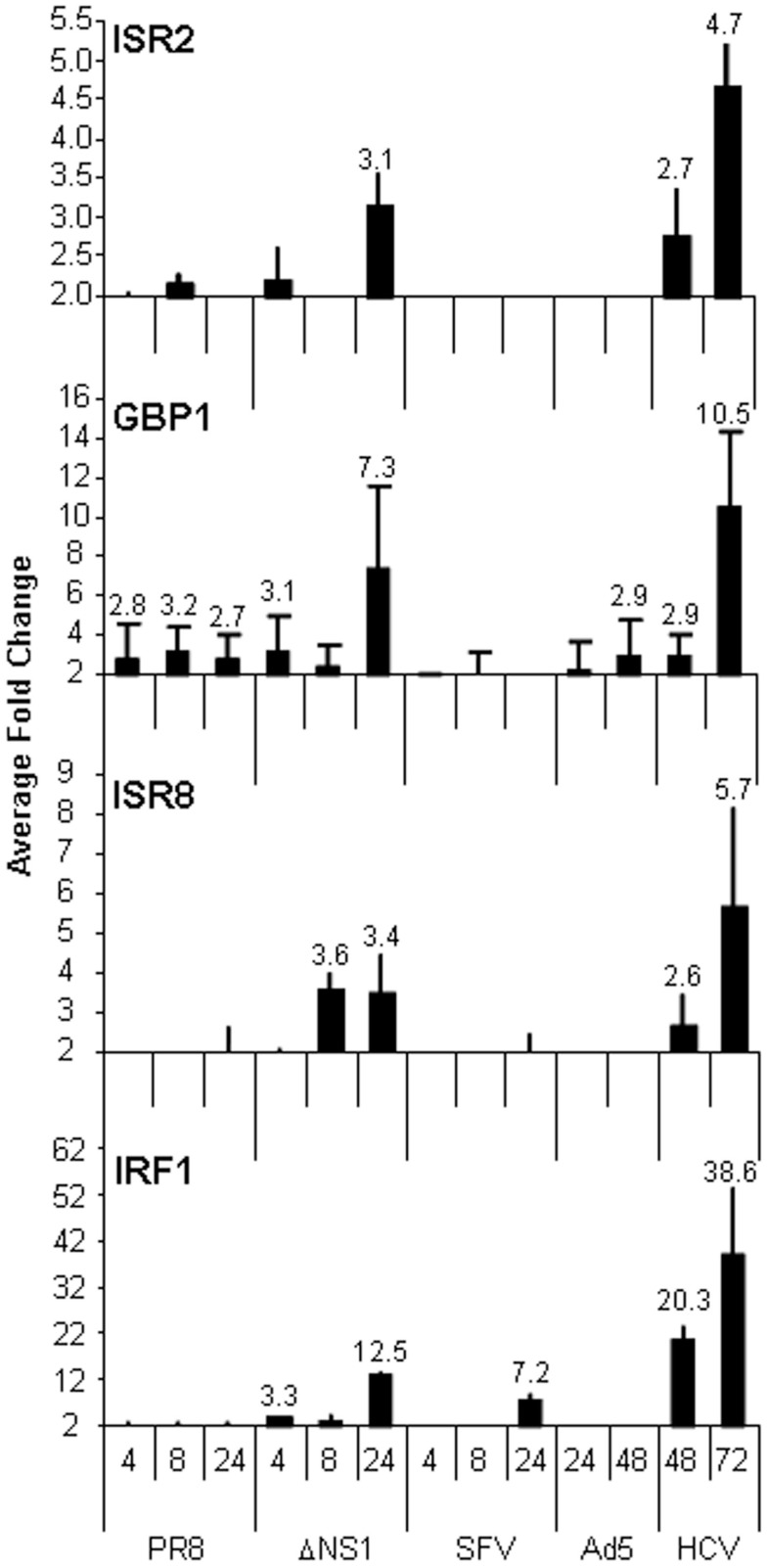
**ISRs respond to viral infections in cultured cells**. HuH7 cells were mock-treated or infected with wild-type influenza virus (PR8) or a mutant that lacks NS1 (ΔNS1), SFV, Ad5, or HCV for the indicated times. RNA was isolated and the expression levels of ISR2, GBP1, ISR8, and IRF1 were evaluated by qRT-PCR. GAPDH expression was also evaluated and used as a reference to calculate the relative levels of each transcript. The experiment was performed three times. The fold-change of infected versus non-infected cells is indicated. Each value shows the average of three replicas from a representative experiment. Error bars indicate standard deviations. The fold-change of treated versus non-treated cells is indicated at the top of each bar when higher than 2.5.

The results obtained in the guilt-by-association analysis led us to hypothesize that ISR2, 8, and 12 could respond strongly to viral infections or to the IFN response induced by viral infections. To study this hypothesis, we evaluated the expression of these lncRNAs in cells infected with Ad5, a DNA virus, or RNA viruses such as influenza virus, SFV, or HCV. All of them have developed mechanisms to block the cellular antiviral response. Influenza virus control of IFN is exerted primarily by the influenza NS1 protein (59). Therefore, we also infected cells with an influenza virus mutant that lacks NS1. All the viruses used, with the exception of HCV, lead to a fast lytic infection that initiates cell death at 24 h post-infection in the case of influenza virus and SFV, or at 48 h post-infection in the case of Ad5. Therefore, several time points post-infection were evaluated in each case. The results show that ISR12 expression was not altered by infection (data not shown). ISR2 and ISR8 expression was only induced in cells infected with the influenza virus unable to control IFN at later times post-infection, when the IFN response is strongest (Figure 8). In general, the induction pattern was similar for GBP1 and IRF1.

**Figure 9:**
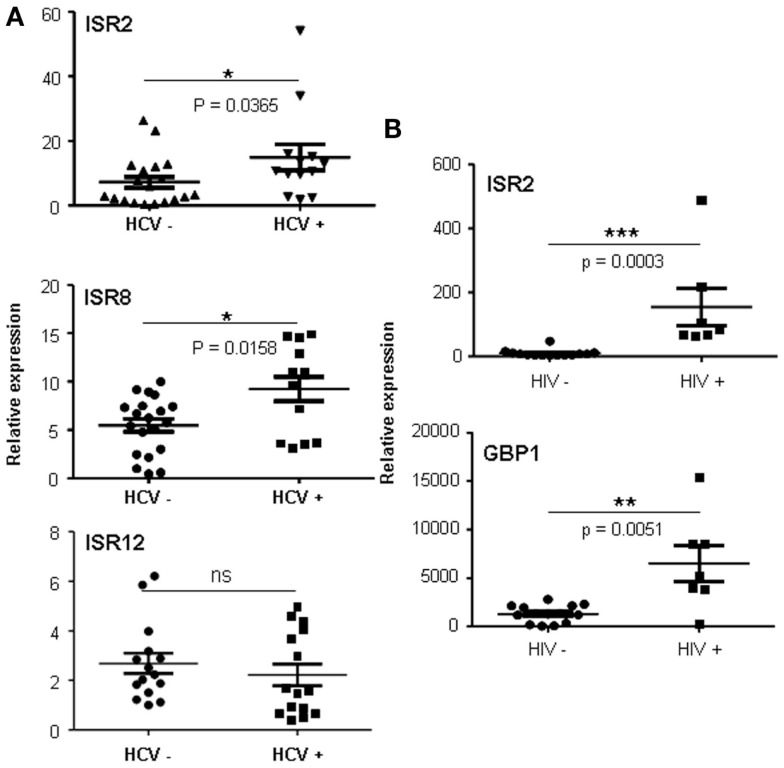
**ISRs respond to viral infections *in vivo***. Expression levels of ISR2 **(A** and **B)**, ISR8 **(A)**, ISR12 **(A)**, and GBP1 **(B)** were evaluated in livers from HCV-negative (*n* = 19) to HCV-positive (*n* = 13) patients **(A)** and in blood cells **(B)** isolated from healthy patients (*n* = 14) or from patients infected with HIV (*n* = 7). Statistical significance was calculated using a two-tailed non-parametric Mann–Whitney*t*-test.

We were surprised to see that the strongest increase in ISR2 and 8 was observed in cells infected with HCV, an IFN-sensitive virus that employs several viral proteins to block the IFN pathway. Increased expression was also observed for GBP1 and IRF1 but not for other ISGs such as OAS (Figure 8 and data not shown). To determine whether a similar upregulation could be observed in HCV patients, levels of ISR2, 8, and 12 were evaluated in livers from HCV-negative (*n* = 19) to HCV-positive (*n* = 13) patients. The results show that both ISR2 and 8 are significantly upregulated in HCV patients (Figure 9A). No differences were observed in the levels of ISR12 in the same samples. Finally, we wanted to determine whether these lncRNAs also respond to other chronic viral infections relevant for human health. Therefore, we evaluated the expression of ISR2, 8, and 12 in blood cells isolated from healthy patients or from patients chronically infected with HIV. We could not detect expression of ISR8 or 12 in these samples. However, both ISR2 and GBP1 were significantly upregulated in HIV-infected patient cells (Figure 9B).

## Discussion

In this work, we show that IFN treatment alters the expression of several lncRNAs in human cells. These lncRNAs were identified in a high-throughput analysis using conditions that detect increased levels of coding genes that respond to IFN both at early or late times (Figures 1 and 2 and Figure S1 in Supplementary Material). In the cells treated with IFN for 3 days, we found, with a very high statistical significance (*B* > 7), an upregulation of well-characterized ISGs such as Mx1, STAT1, IRF9, ISG15, BST2 and several members of the GBP, OAS, and IFI families (Figure 2). Besides, the IFN-induced STAT pathway shows the highest enrichment by Ingenuity analysis (Figure S1 in Supplementary Material). This suggests that high levels of IFN could maintain an active JAK/STAT pathway in HuH7 cells even at late times post-IFN treatment. Therefore, we feel that among the lncRNAs identified in this work, there may be some candidates whose expression is controlled directly by the JAK/STAT pathway, while other candidates could be controlled by other ISGs or by later downstream effectors of the IFN response.

Comparison of the results obtained in the array between coding and lncRNA genes gave an unexpected result: 90% of the altered coding genes but only 54% of the lncRNAs were upregulated by IFN. This suggests that there could be an IFN-mediated repression of genes that affects more strongly lncRNAs. However, even if IDRs showed a generally decreased expression in the presence of IFN (Figure 3B), the downregulation was mild. Validation of downregulated genes showed that only IDR16 was strongly affected by IFN at late times post-treatment (Table S5 in Supplementary Material). Further experiments will be required to determine whether there is a relevant downregulation of lncRNA genes in response to IFN. Notably, the majority of the IDRs match with genomic regions that have not been associated with active transcription in public databases. This is surprising, as some of them such as IDR4, 7, 15, or 16 are expressed at high levels in HuH7 cells according to the qRT-PCR data (Table S5 in Supplementary Material). Therefore, it would be interesting to analyze IFN regulation of lncRNAs using RNASeq, as this may yield a more comprehensive picture of the lncRNA transcriptome and its manipulation by IFN. In fact, during the revision of this paper, another manuscript was accepted showing that transcriptome analysis by RNASeq allowed the identification of several lncRNAs whose expression is altered in response to IFN (82). Similar results have been obtained using RNASeq in our lab (Barriocanal et al., submitted).

Several ISRs were clearly validated by qRT-PCR. Specifically, ISR1, 2, 8, 12, and 22 were upregulated more than fivefold, and ISR2 and ISR8 even more than 50-fold. ISR1 was expressed at very low levels. In contrast, ISR2, 8, and 12 were well expressed in all cell lines tested and their expression was strongly induced by IFN at early (ISR2 and 8) or late times (ISR12) (Figure 4A). Furthermore, low doses of type I IFNα or IFNβ also induced the expression of these ISRs (Figure 5 and data not shown). We did not detect significant induction of these ISRs, GBP1, IRF1, or IL6 with type III IFNλ using doses able to induce other ISGs such as OAS, ISG15, or BST2 (data not shown). Further experiments are required to determine whether these genes could show some specificity for type I IFN, as the repertoire of genes that are induced by type III IFNs is essentially the same as those induced by type I IFNs (83). Finally, ISR12 was also induced after the activation of the NFκβ pathway by TNFα, LPS, or polyI:C (data not shown). This result is in line with the identification of NFκβ binding sites in ISR12 promoter by chIP Seq.

Our molecular and bioinformatic analyses strongly suggest that ISR2, 8, and 12 are indeed long non-coding RNAs. The reasons are: (i) they accumulate preferentially in the nucleus of IFN-treated or untreated cells (Figure 6); (ii) they are marked as lncRNAs with high sensitivity and specificity after analysis of their ORF size and coverage, analysis of their codon and hexamer usage bias, or analysis of PhyloCSF; (iii) they have not been identified as associated with ribosomes in ribosome profiling experiments; and (iv) if they are translated to small peptides, such peptides have not been identified by proteomic analyses (Figure S4 in Supplementary Material).

Given that some lncRNAs regulate the expression of neighboring genes, we searched for the closest coding gene for each ISR or IDR. Interestingly, ISR2, 8, and 12 have neighboring genes related to the IFN response (Table S4 in Supplementary Material, Figure 4B, Figure S2 and S3 in Supplementary Material). ISR2 is in tandem and downstream of GBP6. It is very unlikely that ISR2 results from run-off transcription from GBP6, as GBP6 expression could not be detected in HuH7 cells (data not shown). ISR12 is in tandem and upstream of IL6 while ISR8 is convergent with IRF1. None of the transcripts annotated for ISR2 or ISR12 overlaps with the transcripts annotated for GBP6 or IL6, respectively (Figure S3 in Supplementary Material). However, the region of ISR8 and IRF1 is more complex. While ISR8 does not overlap with its coding neighbor IRF1, the IRF1 gene also transcribes a longer transcript of poor coding capacity called lncIRF1 (Figure 4B, Figure S2A in Supplementary Material). LncIRF1 expression is induced by IFNα to similar levels than ISR8 (Figure S2C in Supplementary Material). As ISR8 is antisense to lncIRF1, they could potentially regulate each other by transcriptional interference or by antisense mechanisms, although only 17 nt of the mature form of ISR8 are antisense to the mature form of lncIRF1 (Figure 4B and Figure S2A in Supplementary Material).

None of the coding/non-coding pairs share the promoter, making it unlikely that they are co-regulated at the transcriptional level, which would otherwise be a way to explain that both are induced in response to IFN. Instead, the promoters of these ISRs seem to be independent. ISR2 is located within the GBP locus, which could be indicative of a general co-regulation in response to IFN. The ISR8 and ISR12 promoters have NFκB binding sites, although only ISR12 is reproducibly induced in response to TNFα under the conditions tested. Furthermore, ISR8 seems a *bona fide* ISG as the promoter has sites for STAT1 and 2 as well as IRF1 and 2. In spite of this, the possibility exists that IFN activation of a coding ISG could result in an unintended recruitment of transcription factors to the promoter of lncRNAs located nearby. We do not think that this is a general phenomenon, as in the microarray or RNASeq analysis, we do not observe that many lncRNAs located close to ISGs are induced after IFN treatment. Besides, if such unintended transcription occurs, we would expect that the expression of ISR2, 8, and 12 should always correlate with the expression of their neighboring coding genes. This, however, is not the case.

Upon further analysis of the results in Figures 1, 4, and 5, we observed a highly significant positive correlation between the expression levels of ISR2 and GBP1, or of ISR8 and IRF1 (Figure 7A). These correlations may reflect the fact that ISR2 and ISR8 are genes induced by IFN at early time points. In fact, the expression of ISR2 and ISR8 also correlated significantly with the expression of IRF1 and GBP1, respectively (data not shown). Therefore, to analyze correlation in a more stringent manner, we compared the expression of ISR2, 8, and 12 with the expression of all the genes represented in the SurePrint G3 microarray using data from 120 human samples. In this case, we only observed a significant correlation for ISR8 and IRF1 (corr = 0.41 and *p* < 10e−0.5), suggesting that these two genes are co-expressed. In fact, the ISR8 promoter has a conserved IRF1 binding site.

Guilt-by-association genome-wide analysis predicts that ISR2, 8, and 12 could function in the IFN pathway and the antiviral response (Figure 7B). Furthermore, they could be involved in viral processes including viral life cycle and viral transcription. In fact, ISR2 and 8 are upregulated at later times post-infection with an influenza virus mutant that lacks NS1, unable to block the IFN response (Figure 8). This suggests that ISR2 and 8 are increased in response to the physiological amounts of IFN secreted by the cells as a consequence of infection. We did not observe significant responses of ISR2, 8, and 12 to other lytic viruses able to block the IFN response. However, both ISR2 and 8 were significantly upregulated in cells infected with HCV compared to controls. This was observed in HCV-infected cells in culture but also in the livers of HCV-infected patients (Figure 9A). Intriguingly, increased levels of ISR2 and GBP1 were also detected in patients chronically infected with HIV (Figure 9B). We did not observe significant correlations between the levels of ISR2 and ISR8 and clinical symptoms, although patients with higher HIV load tend to have higher levels of ISR2 and GBP1 (data not shown).

HCV is a chronic virus that employs several viral proteins to block the IFN pathway (84). However, the ISG expression profile of some patients with chronic HCV infections indicates that IFN is being produced by the infected cells as well as by neighboring cells (85). This may in turn explain upregulation of ISR2 and ISR8. Upregulation was also observed for the neighboring genes GBP1 and IRF1 but not for other ISGs such as OAS (Figure 8 and data not shown). This raises the question why the infection persist in spite of increased levels of antiviral factors? Different viruses are targeted by unique sets of ISGs (86). In the case of HCV both GBP1 and IRF1 have been shown to have antiviral potential (86–88). IRF1 overexpression on its own can activate a similar set of genes as IFN and can lead to a control of the replication of HCV and other viruses (86). Therefore, for infection to persist, the effects of IRF1 and probably other ISGs should be inhibited in infected cells (84). One intriguing hypothesis for future work is that this inhibition is in fact exerted by ISG lncRNA neighbors.

Further experiments will be required to determine whether these ISRs have a proviral or an antiviral role by affecting the function of ISGs. The only preliminary evidence of an anti-IFN role is the highly significant anti-correlation between ISR2/ISR12 and key factors of the IFN pathway found by genome-wide guilt-by-association studies. Similarly, inhibition of a lncRNA located close to viperin, an ISG that also inhibits HCV replication, has been shown to increase the levels of many IFN-inducible genes (82, 89). Therefore, several lncRNAs could act as negative regulators of the IFN pathway. The guilt-by-association study also shows a positive correlation of ISR8/IRF1 and the IFN response, suggesting that ISR8 could have a positive role in the IFN pathway. One possible approach to further dissect the function of these ISRs in the future will be their inhibition via RNAi. However, resistance to RNAi is a common feature of many lncRNAs that locate in the nucleus away from the RNAi machinery or contain poorly accessible structured sequences. An alternative could be the use of gene editing technologies, provided that the function of the targeted lncRNAs is not essential for the cell, which would prevent their complete deletion. Moreover, it may also be feasible to overexpress selected lncRNAs from plasmids or viral vectors, in order to study gain-of-function phenotypes. In the long run, we are optimistic that these and other approaches will help to further delineate the potential role of lncRNAs in the IFN pathway and in the antiviral response.

## Author Contributions

Elena Carnero and Marina Barriocanal designed and performed the experiments, analyzed and interpreted the data, and contributed to the writing of the manuscript; Celia Prior performed some of the experiments; Victor Segura and Elizabeth Guruceaga were in charge of all the bioinformatics analyses; Kathleen Börner and Dirk Grimm prepared and provided the RNA samples from HIV-infected patients; and Puri Fortes conceived the project and the required experiments, provided the budget, interpreted the data, and wrote the manuscript.

## Conflict of Interest Statement

The authors declare that the research was conducted in the absence of any commercial or financial relationships that could be construed as a potential conflict of interest.

## Supplementary Material

The Supplementary Material for this article can be found online at http://www.frontiersin.org/Journal/10.3389/fimmu.2014.00548/abstract

## Footnotes

^1^http://www.ncbi.nlm.nih.gov/geo

^2^http://frodo.wi.mit.edu

^3^http://www.ingenuity.com

^4^https://genome.ucsc.edu/
