# Supplementary material for: Type I Interferon Regulates the Expression of Long Non-Coding RNAs
Source: Front Immunol. 2014 Nov 6;5:548. doi: 10.3389/fimmu.2014.00548 (PMC4222131; doi:10.3389/fimmu.2014.00548)
Supplement: Supplementary file 1 [file Data_Sheet_1.ZIP › Captions for Supplementary Tables and Figures.pdf]

SUPP FIGURES AND TABLES

Supp. Table 1. Clinical data for the HCV-infected patients used in this study. The table shows the age, gender and the date the sample was collected from a liver transplantation (tx) or a hepatectomy (hepa). The virus subtype is also indicated for most of the patients.

Supp. Table 2. Clinical data for the HIV-infected subjects in our study. The table shows the age, gender, CD4+ cell count and viral load of the recruited patients.

Supp. Table 3. Sequences of the oligonucleotides used in this study.

Supp. Table 4. List of putative lncRNAs upregulated (ISRs) or downregulated (IDRs) in response to IFN. The table indicates for each candidate the description of the best probe from the array, the log fold-change (logFC), the statistical significance (B), the position in the genome of the gene that matches the sequence of the probe used in the array (non-annotated, NA, indicates that transcription has not been linked to the sequence of the probe in public databases), name and alternative names of the gene and sense (S) or antisense (AS) orientation (strand). "Next to" indicates the closest coding gene of each transcript within a distance of 100 kb. "None" denotes that the lncRNA is isolated in the genome or the closest gene is another lncRNA. Further information about the neighboring coding gene includes the strand, the relative position to the ISR or IDR (L, left; R, right; In, overlapping) and a classification. The coding – noncoding pairs can be in tandem, convergent or divergent, when their sequences or their promoters do not overlap and they are tail-to-head, tail-to-tail, or head-to-head, respectively. When the coding – noncoding pairs are closer, they can be antisense (AS) when they are in the opposite direction, overlapping (overlap) when they are in the same direction and "shared promoter" when they seem to share the same promoter according to the DNase I hypersensitivity and the histone marks described by ENCODE for that area.

Supp. Table 5. Validation of IDRs and ISRs at different times after IFN treatment. The exact values of Fig. 3 are shown. The relative expression levels are also indicated for each candidate. VL, very low; L, low; M, medium; H, high; and VH, very high. ND denotes non-determined.

Supp. Fig. 1. Ingenuity pathway analysis of genes differentially expressed in response to IFN. The genes upregulated in the array with a B>1.5 have been analyzed using Ingenuity to find pathways and networks significantly enriched. The best outcome has been obtained for the IFN-induced STAT pathway (A) and the TLR / IRF network (B). Genes shown in red are upregulated by IFN.

Supp. Fig. 2. Analysis of ISR8 transcript expression. (A) UCSC representation of ISR8 genomic location and annotated transcripts. CpG islands and H3K27Ac marks are also shown. A number (from 1 to 7) has been assigned to each ISR8 gene transcript in the sense orientation. Location of the primers used to evaluate the levels of each transcript is indicated with a red arrowhead. ISR8 noncoding RNA, IRF1 and lncIRF1 are highlighted in red. Location of the primers that evaluate the expression of lncIRF1 is indicated with a brown arrowhead (B) Expression levels of each ISR8 transcript have been evaluated by qRT-PCR in RNA isolated from HuH7 or HeLa cells treated with 0 or 10000 units/ml of IFNα. (C) Expression levels of ISR8 noncoding RNA, IRF1 and lncIRF1 were evaluated by qRT-PCR in RNA isolated from HuH7 cells treated with 0 or 10000 units/ml of IFNα for 6, 12 or 24 hours. GAPDH expression was also evaluated and used as a reference to calculate the relative levels of each transcript. The fold-change of treated versus non-treated cells is indicated at the top of each bar. The experiment was performed twice and each value shows the average of three replicas from a representative experiment. Error bars indicate standard deviations.

Supp. Fig. 3. Database analysis of ISR2 and ISR12. UCSC representation of ISR2 (A) and ISR8 (B) genomic location and annotated transcripts. The regions of ISR2, GBP6, ISR12 and IL6 are highlighted with a red square to show that they do not overlap.

Supp. Fig. 4. Bioinformatic analysis of the coding potential of ISR2, 8 and 12. The figure shows the results for ISR2 ENST00000513638, ISR8 ENST00000461203 and ISR12 ENST00000435127, but similar results have been observed for other transcripts of the same genes. (A) The analysis with ORF Finder (NCBI) shows all start (green) and stop (pink) codons in the three ORFs (left). The nucleotide position and the peptide length is indicated for each ORF longer than 33 aa (right). (B) Results obtained from CPAT and LNCipedia. ISR2, 8 and 12 have a coding probability and a coding label of "noncoding RNAs" according to these analyses. See the text for details.
