# Supplementary figures and images for: Type I Interferon Regulates the Expression of Long Non-Coding RNAs
Source: Front Immunol. 2014 Nov 6;5:548. doi: 10.3389/fimmu.2014.00548 (PMC4222131; doi:10.3389/fimmu.2014.00548)

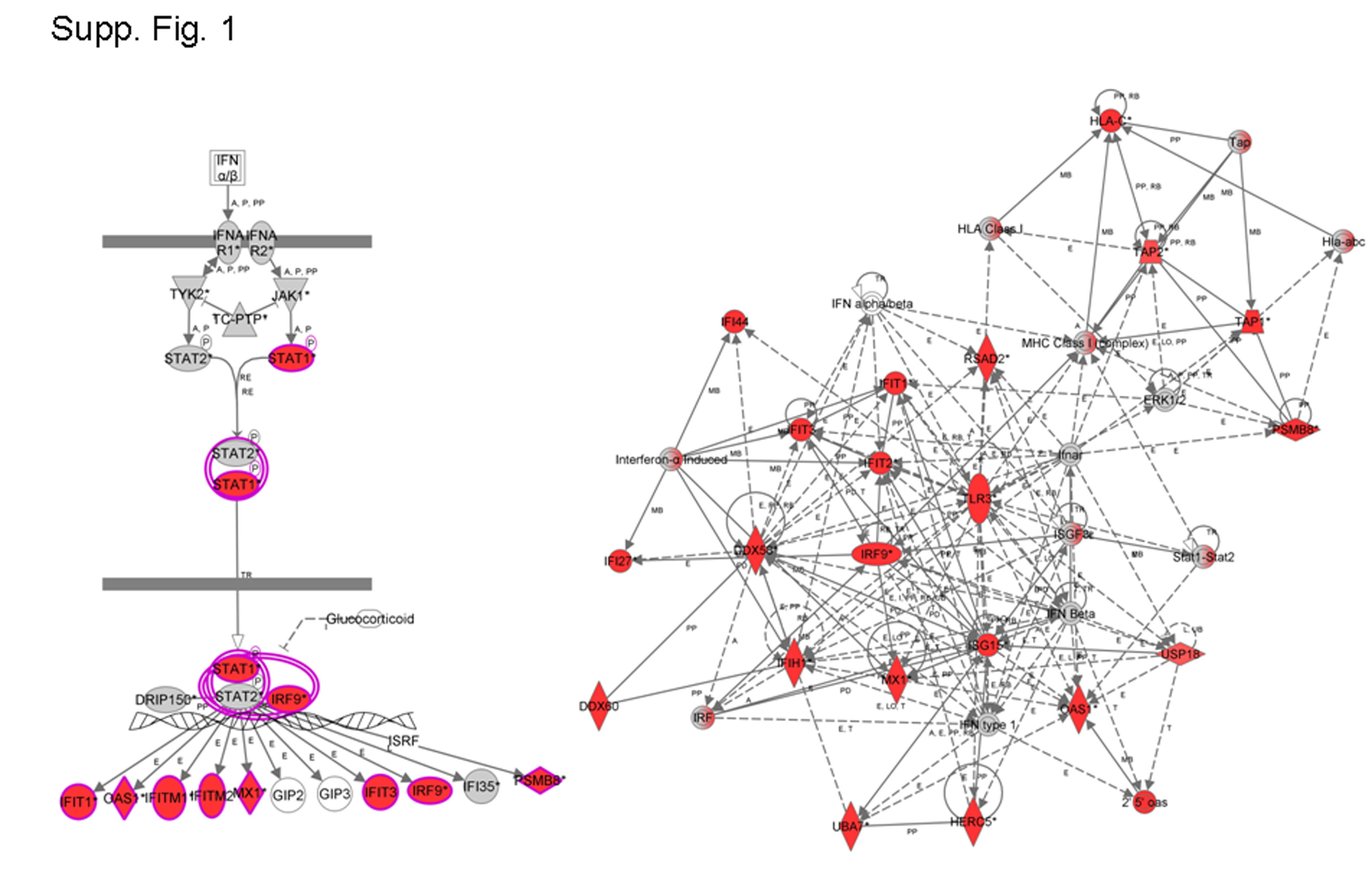

Supplement: Supplementary file 1 [file Data_Sheet_1.ZIP › Figure S1.JPEG]

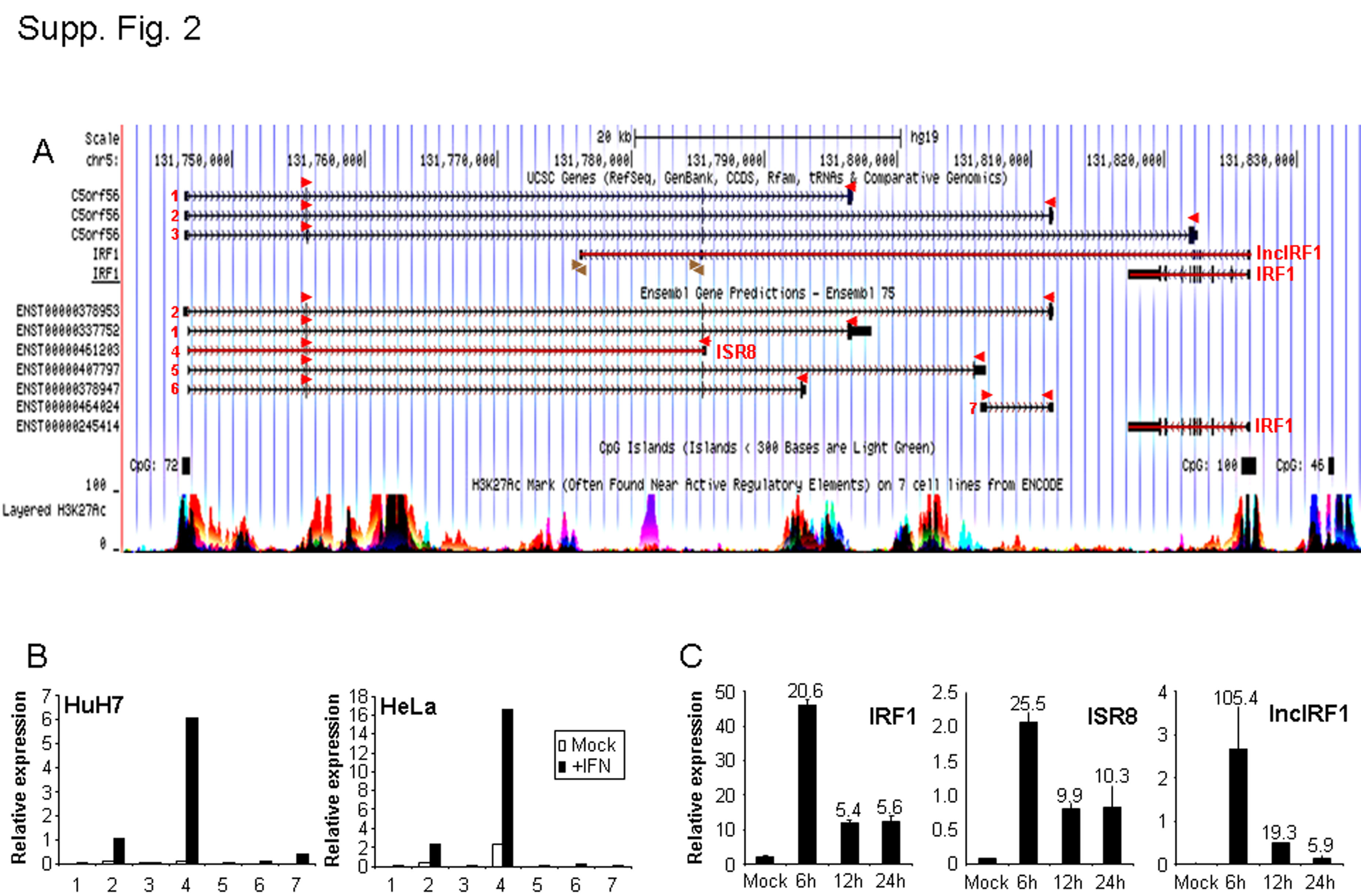

Supplement: Supplementary file 1 [file Data_Sheet_1.ZIP › Figure S2.JPEG]

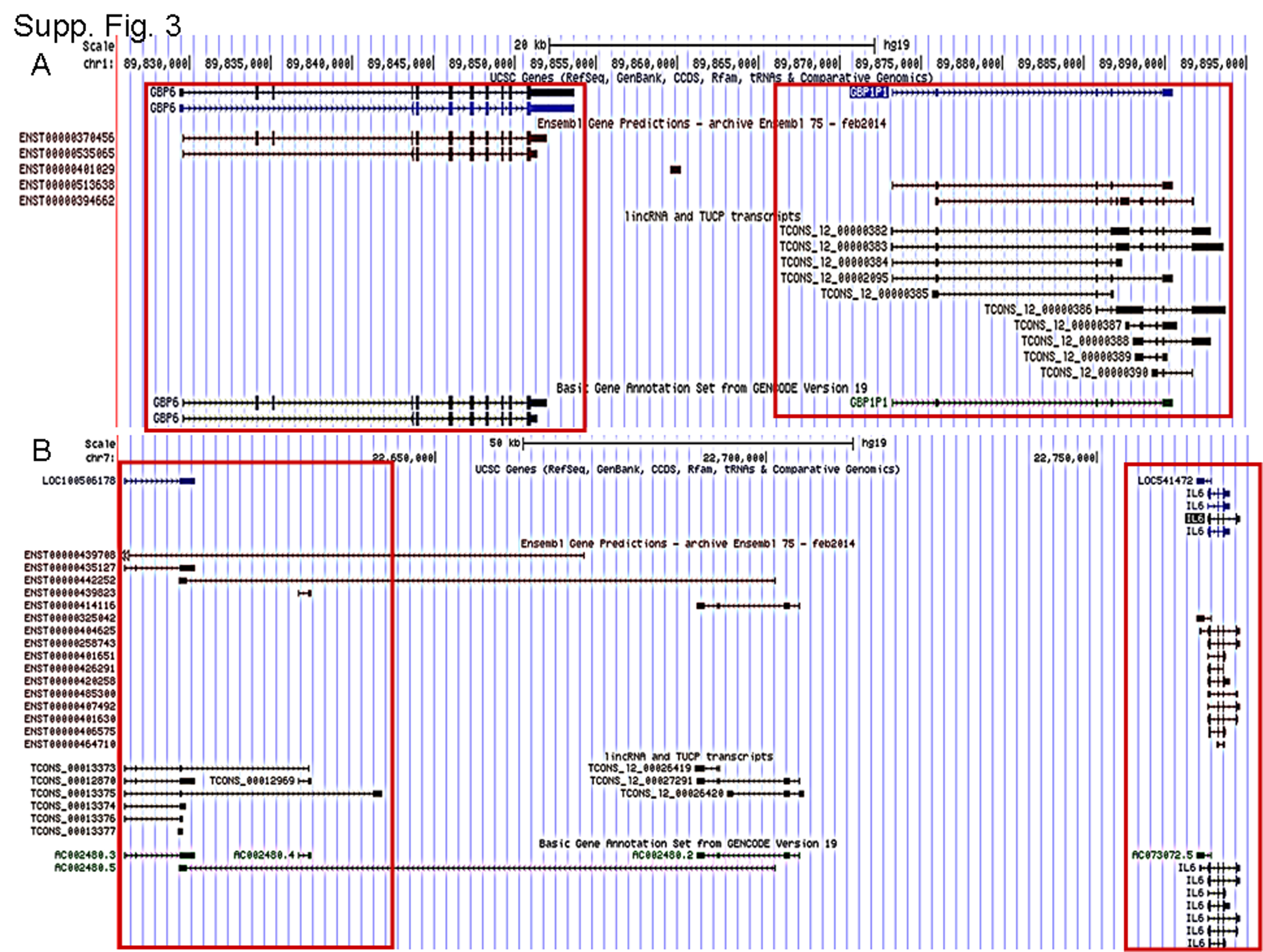

Supplement: Supplementary file 1 [file Data_Sheet_1.ZIP › Figure S3.JPEG]

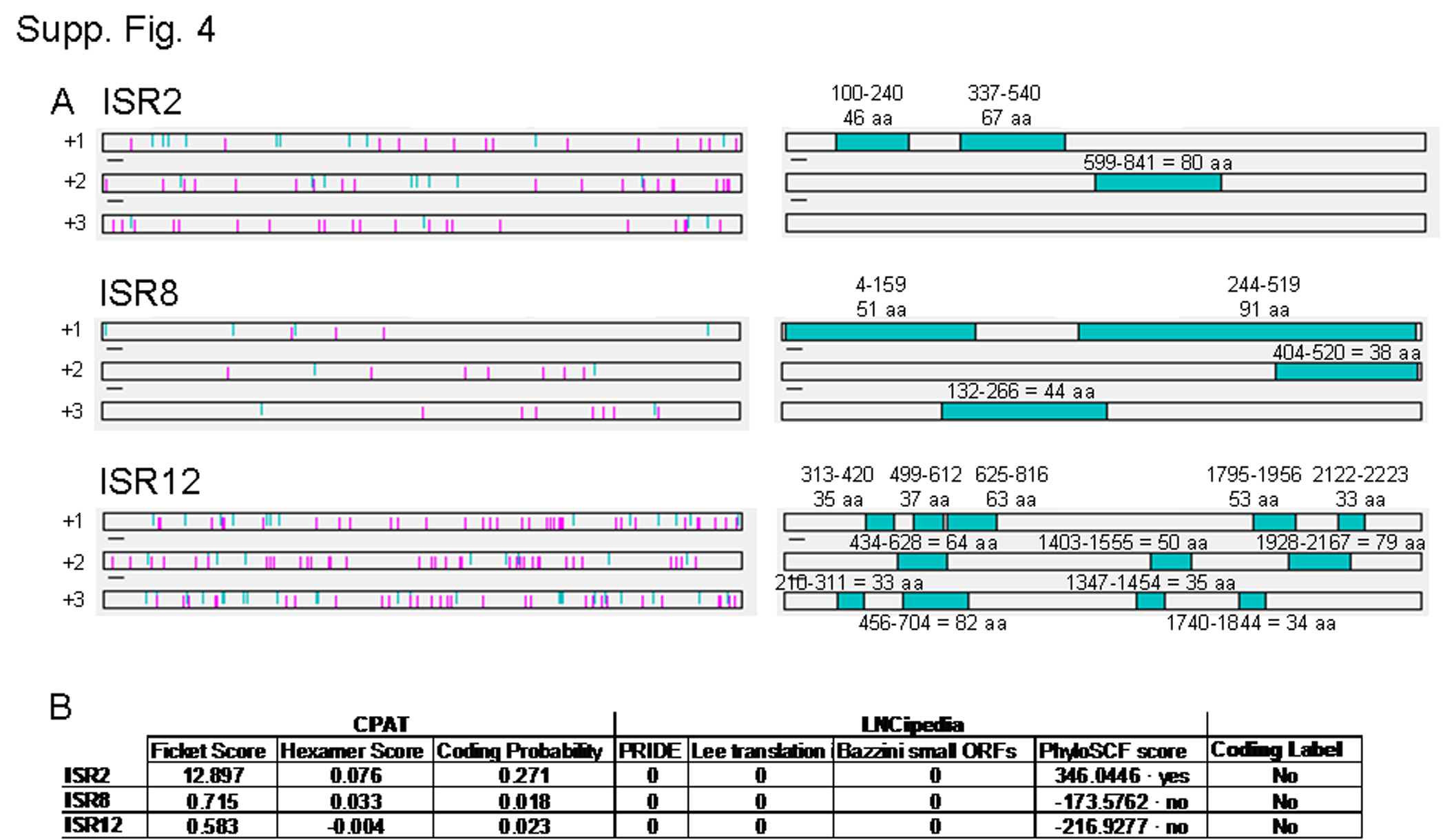

Supplement: Supplementary file 1 [file Data_Sheet_1.ZIP › Figure S4.JPEG]
